# Sprint-Intensity Arm Interval Training May Improve Cardiorespiratory Fitness and Cardiometabolic Health Among Children With Mobility Disabilities: Case Report

**DOI:** 10.2196/64440

**Published:** 2025-10-08

**Authors:** Natalie Dean, Tanvee Sinha, Larsen Bright, Katie M Ellison, Drew Sayer, Raven Young, Drew Davis, James H Rimmer, Byron Lai

**Affiliations:** 1 Department of Physical Medicine and Rehabilitation, University of Alabama at Birmingham, 1600 7th Avenue South 5 Dearth, McWane, Birmingham, AL, 5601, United States, 1 205 638 9790 ext 8; 2 Division of Pediatric Rehabilitation Medicine, Department of Pediatrics, University of Alabama at Birmingham, Birmingham, AL, United States; 3 Division of Family and Community Medicine, Department of Medicine, University of Alabama at Birmingham, Birmingham, AL, United States; 4 Dean's Office, School of Health Professions, University of Alabama at Birmingham, Birmingham, AL, United States

**Keywords:** adapted physical activity, high-intensity interval training, telehealth, teleexercise, cerebral palsy, pediatric rehabilitation, cardiorespiratory fitness, cardiometabolic, case report, aerobic exercise, cerebral palsy, physical disabilities

## Abstract

**Background:**

There are limited options for aerobic exercise options that improve cardiorespiratory fitness and manage cardiometabolic health that are also age-appropriate and suitable for children with mobility disabilities. Children with disabilities require exercise programs that incorporate adapted movements to meet various functional needs, which offer brief training durations to accommodate busy schedules and use remote training methods at home to bypass logistical transportation barriers.

**Objective:**

The aim of this study is to test the potential effects and safety of a sprint-intensity arm-exercise interval training program, combined with music and telehealth, on cardiorespiratory fitness and cardiometabolic health in a child with cerebral palsy.

**Methods:**

This study was a 12-week exercise intervention from pretrial to posttrial for a single child with cerebral palsy (male, age 17 y). The intervention was conducted at the participant’s homes. The participant exercised 3 times per week while following along with YouTube exercise videos. Videos included 4-second maximal sprint bouts followed by periods of rest, which were repeated 30 times during a single exercise session (total of ~2 minutes of maximal exercise). Exercise sessions were supervised by research staff using videoconferencing. Cardiorespiratory fitness was indicated by peak oxygen consumption (pVO_2_), which was measured by a portable metabolic cart during a graded exercise test. Cardiometabolic health outcomes included body composition by dual-energy x-ray absorptiometry scan and a cardiometabolic blood profile by a dried blood spot test. Outcomes were descriptively analyzed.

**Results:**

The participant achieved a 33.6% increase in pVO_2_ (14.6 to 19.5 mL/kg^−1^/min^–1^), a 37.8% improvement in blood triglycerides (82 to 51 mg/dL), and a 15.4% improvement in the total cholesterol to high-density lipoprotein ratio (6.5 to 5.5). Additionally, he had a 5.9% reduction in body weight (171 to 161 lbs) and a 9.6% reduction in total body fat (61.35 to 55.48 lbs) from the arms, legs, and trunk. The participant experienced no adverse events or problems during the intervention. After completing the program, the participant elicited a maximal intensity of exercise using armbands, as demonstrated through pVO_2_.

**Conclusions:**

Sprint-intensity interval training that uses the arms may be safe and potentially effective for enhancing cardiorespiratory fitness and cardiometabolic health in children with physical disabilities. Further research is needed to verify the outcomes of this case report.

## Introduction

Cerebral palsy (CP) is a common motor and physical disability in childhood [1], which can be described as a group of “permanent, non-progressive disorders of the development of movement and posture” and is often accompanied by severe intellectual, conceptual, and social limitations [2]. CP develops from any event that can affect the fetal and neonatal brain and can be categorized by (1) predisposing pathological processes (such as placental vascular disorders), (2) peripartum events (such as chorioamnionitis or abruption), and/or (3) neonatal complications (sepsis, intraventricular hemorrhage) [1]. Although it is not a progressive disease, the clinical presentation of CP can vary as the child grows, requiring ongoing monitoring as well as updated assessments of health risks with subsequent treatments.

Physical impairment increases the difficulty of performing everyday activities, which puts an individual with CP at risk of a sedentary or physically inactive lifestyle [3]. Given their various limitations, individuals with CP have an increased risk of cardiovascular disease, metabolic syndrome, and cardiovascular disease mortality compared to age-matched individuals [4]. Since children with CP who exercise regularly are twice as likely to continue exercising as adults, there is an urgent need to find ways to promote exercise adoption in youth to reduce the health risks associated with this condition as they grow older [5]. Notably, one-third of children with CP are nonambulant and rely on wheelchair mobility [6], and the remaining two-thirds have varying degrees of impairments primarily affecting the lower extremities, which significantly impacts their ability to walk [7]. This makes conventional exercise modalities like running, cycling, and walking generally not feasible for a large portion of people with CP. Currently, there are few proven, evidence-based options for aerobic exercise other than wheelchair propulsion or arm ergometry for nonambulatory individuals or those who require accommodations for their lower limb limitations [8-10].

Sprint interval training (SIT) is a form of high-intensity interval training (HIIT), which is a time-efficient and evidence-based approach to increase cardiorespiratory fitness through its structure of sets of short “sprints” of activity, with periods of rest in between [11]. SIT involves repeated maximal efforts typically lasting less than 30 seconds, designed to reach near-maximal intensity in short bursts [12,13]. In contrast, HIIT consists of intervals performed at submaximal intensities around 80%‐95% of an individual’s maximal heart rate, with longer activity durations [12,13]. Compared to HIIT, SIT may be more practical for this population since it produces significant metabolic and muscular adaptations with seemingly less overall physiological stress [13,14]. SIT uses primarily anaerobic type II fast-twitch muscle fibers and features shorter exercise intervals, which in theory allows for greater replenishment of anaerobic energy pathways compared to HIIT [15,16]. Considering that SIT using the arms has been found to elicit similar benefits to programs that use the legs due to musculature differences [17], a SIT arm program may be ideal for people with mobility disabilities. Additionally, access to the program can be enhanced by combining telehealth technology [18], which bypasses barriers related to transportation and a lack of nearby accessible facilities for people with disabilities [19].

This study modified an existing movement-to-music telehealth program [20] to incorporate SIT training using the arms for one adolescent with CP. The previous program included long durations of low-to-moderate intensity exercises using rhythmic movements to music, along with adult actors. This study aimed to test a SIT program with brief exercise sessions to make it more convenient for families to perform, combined with child-appropriate video themes and music. To the best of our knowledge, interventions related to this form of training have not been published previously.

The purposes of this study were to:

1. Examine the potential effects of the movement-to-music SIT program on the health of a child with CP. Health outcomes included cardiorespiratory fitness, body composition, and cardiometabolic health.
2. Evaluate the safety of a SIT program in a person with CP through adverse events and problems.
3. Evaluate the intensity of exercise that can be achieved from SIT by analyzing oxygen consumption during exercise.

## Methods

### Recruitment of Participant

The participant included in this study was a member of the research team. Eligibility criteria included wireless internet access at home, being between the ages of 10 and 17 years, a medical diagnosis of CP, the ability to exercise using the arms, self-reported mobility disability, and a caregiver to support the study procedures of the child. The participant recruited for this study was a 17-year-old male with spastic diplegic CP. He had mild-to-moderate hemiparesis, intellectual disability, and ambulated independently (Gross Motor Function Classification System level II). He received regular botulinum toxin injections for the management of spasticity in his lower limbs.

### Ethical Considerations

The case study was not considered research by the university review board; thus, review and approval by the board was not required. As per university guidelines for case studies, written informed consent and assent were still obtained from the participant and their caregiver prior to participation. Study data were deidentified using alpha-numeric codes, and participation was confidential. No compensation was provided for participation. No artificial intelligence was used in any portion of the manuscript.

### Procedures

The participant came to the laboratory for a baseline data collection visit, participated in the 12-week intervention, and then came back to the laboratory for a post–data collection visit. Health outcomes were measured predata and postdata. On the postvisit, he conducted an evaluative exercise assessment for our third study purpose. The participant completed the intervention in May 2022. This case study informed the development of a pilot randomized controlled trial [21].

### Intervention

**Figure 1.**
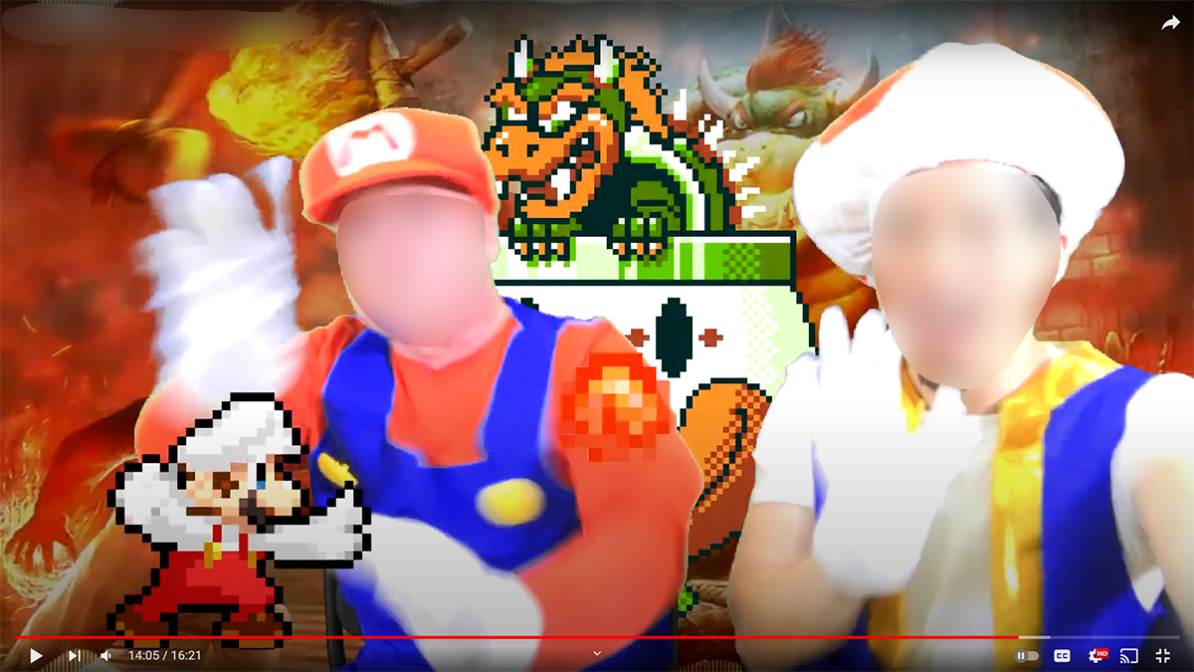
Demonstration of a sprint-intensity arm interval training video used in a 12-week, home-based, upper-body movement-to-music program designed to make high-intensity exercise more accessible and enjoyable for children with mobility disabilities. The program was performed by a 17-year-old male participant with spastic diplegic cerebral palsy.

The intervention prescription included a 12-week SIT program. The SIT program included 3 sessions per week that each lasted 15‐25 minutes. Each SIT session included a warm-up followed by 30 repeated sequences of 4 seconds of all-out arm exercise and 30 seconds of rest (totaling 2 minutes of all-out exercise over the entire session), as well as a cooldown. Rest periods included active or passive recovery that progressed from 30 seconds in week 1 to as low as 16 seconds in week 7 of the program, guided by the participants’ needs. The exercise prescription was based on a previous SIT study [16]. Regarding specific movements, the warm-up primarily included low-intensity repetitive movements that were opposite to those used in the exercise sequence. For example, backward “arm jogging” (shoulder circles backward emphasizing the posterior deltoids) was a common warm-up movement, given that most exercise movements used the anterior deltoids. Exercise movements coincided with the theme of the exercise video. Movements included gross shoulder and elbow movements, such as climbing motions, rope swinging, lightsaber chopping, web shooting, and vertical and straight forward punching. Videos informed the viewer to mirror or modify the movements to what felt “good” to them (eg, use only arm). Videos included verbal cues to encourage high-intensity exercise through verbal cues: “Try your very best!” “Don’t hold back,” and “Use all of your energy.” Videos were based on age-appropriate themes, specifically, 3 popular fictional superhero genres: Spider-Man, Star Wars, and Super Mario. The videos included 2 actors, 1 disability exercise expert (BL), and 1 child with CP (LB; Figure 1).

Each session was guided by a video that was played from a cloud-based server (YouTube). The intervention used a web-based platform, Zoom (Zoom Communications Inc), to allow videoconference supervision by a research staff member who was stationed at the research laboratory. The participant wore a Garmin Vivosmart 3 activity monitor (Garmin), which was used for a gross approximation of heart rate during exercise. Periodically during exercise, the research staff member would ask the participant what their heart rate reading was on their activity monitor, as well as their rating of perceived exertion using a 0‐10 scale [22]. At the end of each session, the participant was asked to rate their perceived exertion to gauge fatigue from the session as a whole. If exertion was lower than a score of 7 for 3 consecutive sessions, resistance bands were to be introduced as a form of exercise progression. Resistance bands were added in the tenth week of the program. The participant was consistently asked to keep his diet and nutrition habits stable throughout the intervention period.

### Exercise Intensity Evaluation Procedure

At the postvisit, the participant was provided with 20 minutes of rest after his graded exercise test, after which he underwent an exercise evaluation of the SIT program. For the evaluation, the participant was equipped with the portable metabolic cart and exercised along with one of the SIT videos taken from the later weeks of the program (ie, a video with less rest). The purpose of this was to assess whether arm SIT could elicit a high intensity of exercise. According to the American College of Sport Medicine guidelines for determining exercise intensity, low intensity is characterized as 57%‐63% of the age-predicted maximum heart rate, moderate as 64%‐76%, vigorous as 77%‐95%, and near maximal or maximal as ≥96%, respectively [23]. In addition to the American College of Sports Medicine guidelines, we utilized the Tanaka equation (208 – 0.7 × age) for live target heart rate zones during exercise [24]. For the evaluation, the participant was asked to rest for 5 minutes, perform a warm-up of 3 minutes, and then do 3 stages of arm SIT: 3 minutes with no resistance band, followed by a 1-minute rest period, 3 minutes with a low-grade resistance band and a 1-minute rest period, and then 3 minutes with a midgrade band. Oxygen consumption was measured continuously throughout the evaluation.

Adverse events were monitored and recorded during the supervised sessions by the exercise coach. Additionally, during each call, the participant was asked how they were feeling since their last session, with a specific focus on whether their shoulders or arms were too sore to begin exercise and whether they experienced any injuries or problems since their last session.

### Measures

Outcome measures were obtained before and after the 12-week program (at weeks 0 and 13). The primary outcome of interest was cardiorespiratory fitness, indicated by peak oxygen consumption (pVO_2_), which was measured by a portable metabolic cart (COSMED K5, COSMED) during a graded exercise test using an arm ergometer. The test increases in resistance, and the individual performs the exercise until they can no longer continue. Test values were expressed as milliliters per kilogram per minute (relative measure).

Cardiometabolic health measures included a dual-energy x-ray absorptiometry scan to evaluate body composition (total mass, total lean mass, percent lean tissue, and percent fat tissue), a dried blood spot test to evaluate the lipoprotein profile (triglycerides, cholesterol, and low-density and high-density lipoprotein), glucose and glycated hemoglobin A_1c_ tests, and body weight measurement using a scale. To evaluate safety, the research staff consistently monitored the participant for adverse events (eg, muscle soreness/injury, overexertion, and illness).

## Results

### Feasibility Data

The participant completed all 36 prescribed exercise sessions (100% attendance) with no adverse events or problems. An exercise band was introduced in week 10 to maintain an increased level of exertion. Heart rate monitoring of exercise intensity did not work as planned and was not recorded during the sessions, despite being planned. The data did not appear accurate for two reasons: (1) the participant was taking medications that blunt heart rate and (2) there was a notable delay between stopping the exercise and the participant reporting the heart rate to the coach (primarily due to difficulty reading the small values on the wrist monitor because the participant had substantial visual impairment).

### Health Outcomes

At baseline, the participant had a BMI of 31.4 kg/m^2^ and dyslipidemia (high-density lipoprotein <40, elevated total cholesterol:high-density lipoprotein ratio; Table 1). The participant achieved a 33.6% increase in pVO_2_ (14.6 to 19.5 mL/kg^−1^/min^–1^), a 37.8% improvement in blood triglycerides (82 to 51 mg/dL), and a 15.4% improvement in their total cholesterol to high-density lipoprotein ratio (6.5 to 5.5). Glucose and glycated hemoglobin A_1c_ remained stable (88% to 80% and 5.2% to 5.1%, respectively). Additionally, he had a 5.9% reduction in body weight (171 to 161 lbs), and a 9.6% reduction in total body fat (61.35 to 55.48 lbs) from the arms, legs, and trunk (14.9%, 9.6%, and 8.72% reduction, respectively).

**Table 1.** Pre and post outcomes.

Outcome	Baseline	Postintervention	Percent change
BMI (kg/m^2^)	31.4	29.6	–5.7
Dyslipidemia (high-density lipoprotein <40; total cholesterol:high-density lipoprotein ratio)	6.5	5.5	–15.4
Peak oxygen consumption (mL/kg/min)	14.6	19.5	33.6
Blood triglycerides (mg/dL)	82	51	–37.8
Glucose (mg/dL)	88	80	–9.1
Glycated hemoglobin A_1c_ (%)	5.2	5.1	–1.9
Body weight (lbs)	171	161	–5.9
Total body fat (lbs)	61.35	55.48	–9.6
Arm fat (%)	34.9	30.3	–13.2
Leg fat (%)	39.4	38.8	–1.5
Trunk fat (%)	40.9	39.6	–3.18

### Exercise Intensity Evaluation

**Figure 2.**
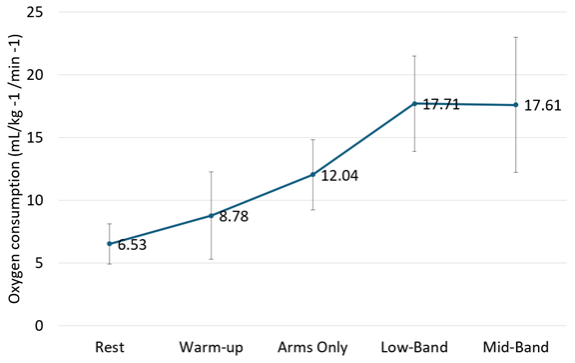
Mean oxygen utilization across graded sprint-intensity arm intervals in a 17-year-old male with spastic diplegic cerebral palsy. Increases in resistance (no, low, or medium) led to near-maximal oxygen uptake, demonstrating the potential for high-intensity cardiovascular output. Error bars indicate SD.

Results for the exercise evaluation are shown in Figure 2. At rest, the participant had a pVO_2_ of 6.53 (SD 1.6) mL/kg^−1^/min^−1^. During warm-up, he had a pVO_2_ of 8.78 (SD 3.5) mL/kg ^−1^/min^−1^. In stage 1 (no band), he exercised at a moderate intensity of 61.8% of his pVO_2_ (12.04/19.5 mL/kg^−1^/min^−1^). In stage 2 (low-grade resistance band), he exercised at a near-maximal intensity of 90.8% pVO_2_ (17.71/19.5 mL/kg^–1^/min^–1^). In stage 3 (midgrade resistance band), he exercised at a near-maximal intensity of 90.8% pVO_2_ (17.61/19.5 mL/kg^–1^/min^–1^) [19]. Visually, the participant was performing faster movements during stage 2 (low-grade band) than he was in stage 3 (midgrade band). The rating of perceived exertion was 3 for the warm-up, 7 for exercise with no resistance band, and 10 at the end of each band stage.

## Discussion

This case study tested a unique arm-exercise protocol as a method for cardiovascular exercise that could be used by children with mobility disabilities. The 12-week protocol included repetitive maximal-intensity arm movements in short intervals (4 s on and 16‐30 s rest), which resulted in a brief exercise duration for each session. The study aimed to explore the potential benefits of SIT exercise on indicators of cardiometabolic health. This case study demonstrated that SIT may be a potentially beneficial method for improving cardiovascular and cardiometabolic health among children with mobility disabilities. Additionally, the study suggests that even an experienced exerciser who completed 12 weeks of the intervention can achieve near-maximal intensities of exercise while following along with a SIT music video.

The participant experienced a 33.6% increase in pVO2. This increase was slightly lower than what has been observed from SIT using the arms in the general adult population [17]. However, this was slightly higher than the 18%‐22% that has been observed from short-term aerobic exercise studies conducted with children with CP [25]. Additionally, this study identified benefits to cardiometabolic health, namely reduced body weight and improved body composition, as well as some health-related blood biomarkers. Improvements to cardiometabolic health are critical, considering that few studies have demonstrated improvements to cardiometabolic health, particularly from seated arm exercises [9]. These findings are important considering that children with disabilities are at a two- to three-fold risk of mortality from cardiovascular disease and increased risk of metabolic syndrome compared to age-matched individuals secondary to physical inactivity and higher prevalence of dyslipidemia, hypertension, and fatigue [26,27]. In a recent population-based study, 17% of people with CP were classified as having metabolic syndrome and were at a 20%‐40% increased risk for cardiovascular disease (39.7% BMI-based and 26.55% lipid-based) [4]. Nevertheless, further research is necessary to begin to confirm these findings, considering that case studies typically have larger effects than randomized controlled trials.

A strength of the intervention is that it can be widely distributed through video cloud servers (like YouTube), allowing for easy replication and implementation in a large randomized controlled trial.

As this was a case study, there were several limitations. First, the findings should be interpreted with caution, considering that there was a single participant. Second, the participant was a member of the study team, which exposes the findings to a high level of bias. Third, we found that measuring exercise intensity using a wrist-worn monitor was not suitable because of heart rate blunting medications taken by the participant and difficulties with obtaining a real-time heart rate through verbal communication using the web-based platform (due to a long delay in the participant checking their watch and reporting the result to the coach). Fourth, the participant’s outside physical activity participation and nutritional habits were not monitored, which could have confounded the results.

This study identified a convenient and potentially beneficial protocol for improving cardiorespiratory and cardiometabolic health among children with CP. As the intervention uses exercise videos with minimal supplies and only arm exercises, it is a potentially scalable intervention for a large population of children with mobility disabilities. However, further research is warranted to examine the effects of arm SIT with confirmatory sample sizes and study designs.

## Abbreviations

CP: cerebral palsy
HIIT: high-intensity interval training
pVO_2_: peak oxygen consumption
SIT: sprint interval training
